# Predators' decisions to eat defended prey depend on the size of undefended prey^☆^

**DOI:** 10.1016/j.anbehav.2013.03.021

**Published:** 2013-06

**Authors:** Christina G. Halpin, John Skelhorn, Candy Rowe

**Affiliations:** a Centre for Behaviour and Evolution, Newcastle University, Newcastle upon Tyne, U.K.; b Centre for Research in Animal Behaviour, University of Exeter, Exeter, U.K.

**Keywords:** aposematism, educated predator, energy, European starling, foraging, mimicry, prey size, *Sturnus vulgaris*, toxic prey

## Abstract

Predators that have learned to associate warning coloration with toxicity often continue to include aposematic prey in their diet in order to gain the nutrients and energy that they contain. As body size is widely reported to correlate with energetic content, we predicted that prey size would affect predators' decisions to eat aposematic prey. We used a well-established system of wild-caught European starlings, *Sturnus vulgaris*, foraging on mealworms, *Tenebrio molitor*, to test how the size of undefended (water-injected) and defended (quinine-injected) prey, on different coloured backgrounds, affected birds’ decisions to eat defended prey. We found that birds ate fewer defended prey, and less quinine, when undefended prey were large compared with when they were small, but that the size of the defended prey had no effect on the numbers eaten. Consequently, we found no evidence that the mass of the defended prey or the overall mass of prey ingested affected the amount of toxin that a predator was willing to ingest, and instead the mass of undefended prey eaten was more important. This is a surprising finding, challenging the assumptions of state-dependent models of aposematism and mimicry, and highlighting the need to understand better the mechanisms of predator decision making. In addition, the birds did not learn to discriminate visually between defended and undefended prey based on size, but only on the basis of colour. This suggests that colour signals may be more salient to predators than size differences, allowing Batesian mimics to benefit from aposematic models even when they differ in size.

## Highlights

• The size of toxic prey did not affect the amount of toxin ingested by birds.
• Total prey mass eaten did not affect the amount of toxin ingested by birds.
• The amount of toxin ingested by birds depended on the mass of nontoxic prey.
• Colour signals may be more salient to predators than size differences.
• Findings have implications for the selection pressures acting on aposematic prey.

Aposematic insects advertise their toxicity to potential predators with conspicuous coloration or markings (Wallace 1867; Poulton 1890; Cott 1940). Naïve predators readily learn to associate the visual warning signal with toxicity and reduce their attacks on aposematic prey (Friedlander 1976; Gittleman et al. 1980; Guilford 1988; Alatalo & Mappes 1996). However, at the end of the learning process, predators may still include some aposematic prey in their diet (e.g. Brower & Calvert 1985; Chai 1986; Pinheiro 1996; Skelhorn & Rowe 2006; Halpin et al. 2012), even when they know that these prey contain toxins (e.g. Barnett et al. 2007; Skelhorn & Rowe 2010; Halpin et al. 2012). This is because educated predators benefit from eating the nutrients and energy that aposematic prey contain, particularly when they are in a poor energetic state (Sexton et al. 1966; Williamson 1980; Chai 1986; Hileman et al. 1995; Barnett et al. 2007, 2012). Therefore, educated predators make decisions to eat aposematic prey based on the trade-off between the benefits of gaining nutrients and energy with the costs of ingesting toxins (Barnett et al. 2007; Skelhorn & Rowe 2007, 2010). Understanding how predators make these decisions is important because predators can exert different selection pressures on aposematic prey when they are educated compared with when they are naïve, significantly altering the evolutionary dynamics of aposematism and mimicry (Kokko et al. 2003; Sherratt 2003; Sherratt et al. 2004).

Although there have been a number of recent studies investigating how the physiological state of a predator (particularly its energetic state) affects its decisions to eat aposematic prey (e.g. Hileman et al. 1995; Barnett et al. 2007, 2012), the energetic value of the aposematic prey itself has rarely been considered (Brower & Calvert 1985; Turner & Speed 2001; Sherratt 2003). There is a clear prediction that if two species of aposematic prey are equally toxic, predators should prefer to eat the most valuable species: the one that provides the most nutrients and energy in order to maximize the nutritional benefits relative to the costs of ingesting the toxin. In addition, any additional energy and nutrients acquired by eating larger prey could potentially allow predators to invest more in detoxification processes, which in turn would enable a predator to ingest more toxic prey (Villalba & Provenza 2005; Dziba et al. 2007). However, to our knowledge, no study has tested the effects of the energetic content of toxic prey on the foraging decisions of predators, in order to investigate how it affects selection pressures acting on aposematism and mimicry.

In this experiment, we used body size as a proxy for the energetic content of aposematic prey. Body size is a strong predictor of the amount of nutrients and energy that prey contain (e.g. Wiegert 1965; Griffiths 1977; Barnard & Brown 1981; Barnard & Stephens 1981), and predators have strong preferences for undefended prey that are larger (Barnard & Brown 1981; Brower & Calvert 1985) or for sizes that maximize their rates of energetic gain (e.g. Pyke et al. 1977 and references therein; Stephens & Krebs 1986). Thus, we would expect predators to increase their ingestion of an aposematic prey type when prey individuals are large rather than small. Notably, increasing the body size of aposematic prey could also increase the size of the aposematic signal, which can affect both the detectability and the efficacy of the signal (Hagman & Forsman 2003; Nilsson & Forsman 2003; Mänd et al. 2007; Sandre et al. 2007; Remmel & Tammaru 2009, 2011). Therefore, in our experiment, we manipulated the body size of the aposematic prey while keeping the colour signal a constant size in order to investigate specifically the effect of changes in body size and energetic reward, and not changes in signal detectability.

Although we predicted that the body size of toxic prey would affect the foraging decisions of predators, it is also likely that in nature these decisions will be influenced by the energetic content of alternative, undefended prey in the environment. In fact, predators are expected to eat more defended prey when the availability of energy from alternative palatable prey is low (Brower et al. 1968; Turner & Speed 2001; Kokko et al. 2003; Sherratt 2003), but there has been equivocal support for this idea (Lindström et al. 2004; Rowland et al. 2010). However, previous experiments have measured the effects of undefended prey abundance on how naïve predators learn to avoid aposematic prey, and not specifically tested how educated predators make energy–toxin trade-offs and decisions about prey when the energetic value of the aposematic prey changes. In our experiment, we manipulated the size rather than the abundance of the palatable prey, in order to control carefully the energy available from undefended prey and provide the first direct test of the hypothesis that the predation of toxic prey will increase when the energy content of alternative prey decreases.

We used a well-established experimental system, presenting wild-caught starlings, *Sturnus vulgaris*, with randomized sequences of undefended and defended mealworms, *Tenebrio molitor* (e.g. Skelhorn & Rowe 2006, 2007, 2009), to test how prey size affects the foraging decisions of educated predators. We were able to measure not only the numbers of defended and undefended prey eaten, but also the prey mass and the amount of toxin eaten by each bird to understand better what factors affect how educated predators make decisions about energy–toxin trade-offs. We predicted that birds would increase their ingestion of defended prey when the relative energetic content of these prey was increased, either by alternative undefended prey being small or by the defended prey being large and thus: (1) birds would eat more of the defended prey when they were large compared with when they were small; and (2) more defended prey would be eaten when undefended prey were small compared with when they were large.

## Methods

### Subjects and Housing

Forty (17 males, 23 female) wild European starlings were caught using a whoosh net, in October 2011 (outside of the breeding season), under licence (English Nature 20093299). When caught, birds were placed individually in cloth bird bags for transport purposes. Upon arrival at the laboratory, and before being released, all birds were checked by a veterinarian. The birds were kept in two indoor free-flight aviaries (20 birds per aviary), measuring 215 × 340 cm and 220 cm high, which were enriched with natural tree branches, water baths, perches and trays containing bark. They were given chick crumbs, fresh fruit, mealworms and Orlux insect patee (Dietec UK, www.dietec.co.uk) every day. When the experiment was carried out; subjects were housed in pairs in enriched cages measuring 150 × 45 cm and 45 cm high. The birds were given the same diet as when they were in free flight, except that they only received live mealworms during the experimental sessions. Coloured plastic leg rings were used to allow identification of each bird, and all birds were weighed and monitored weekly for welfare purposes. Before an experimental session an opaque plastic divider was put in place down the centre of each cage, effectively producing two cages, each housing one individual from each pair of birds. Each bird was thus visually isolated from its cage mate. On each side of the cage there was a drawer measuring 45 × 75 cm, with a spring-loaded flap facing the front through which prey could be presented. Water was available at all times and food was available ad libitum, except when birds were deprived of food for 1.5 h before a session. After the experiment all birds were returned to free-flight aviaries before being ringed using British Trust for Ornithology rings and released in May 2012 at the same site from which they were caught. Food was placed at the site upon release. All experiments were conducted under Local Ethical Committee approval (ERC Project ID: 266).

### Prey

We weighed mealworms before presenting them to the birds and classified them according to their mass as large (0.22–0.25 g), medium-sized (0.19–0.21 g) or small (0.15–0.18 g) prey. Over this size range, mealworms have a similar energetic content per unit mass (J/kg), and therefore the energetic content of the mealworms that we used increased with size (Finke 2002). Given that the daily energy expenditure of a starling in the laboratory is reported to be around 138 072 J (33 kcal; Brenner 1965; Thomson & Grant 1968), we estimated that our birds would need to eat 43% more of the small undefended mealworms than the large mealworms (97 small mealworms versus 68 large mealworms) to gain their required amount of energy for a day (calculations based on Finke 2002 and the average weight of small and large mealworms used in our experiment). Therefore, we considered the difference in mass and energetic content between our small and large mealworms to be sufficiently large to affect the foraging decisions of the birds. Defended mealworms were injected through the mouthparts, using a hypodermic needle, with 0.02 ml of a 4% quinine solution (made using 4 g quinine sulphate powder dissolved into 100 ml water). Quinine has been used widely as an aversive stimulus in learning experiments (e.g. Alcock 1970; Alatalo & Mappes 1996; Méry & Kawecki 2003; Halpin et al. 2008), and previous work has shown that it cannot be tasted when injected into mealworms in this manner (Skelhorn & Rowe 2009, 2010). Undefended prey were injected with 0.02 ml water.

### Training Sessions

Before all training and experimental sessions, birds were food deprived for 1.5 h to facilitate foraging. Five minutes before the start of a session, a white curtain was erected in front of the cage to isolate the birds visually from other birds in the room and the experimenter. The birds were then observed via video cameras linked to television monitors. During training sessions birds were trained to eat single medium-sized mealworms out of petri dishes (diameter 60 mm). Each bird was given two training sessions on consecutive days; both trials consisted of a series of 24 unmanipulated mealworms presented singly in clear petri dishes. One presentation was made every 3 min. The birds were given 1 min to attack each mealworm, after which the petri dish was removed. If a mealworm was eaten the empty dish was removed immediately. By the second session all birds were eating all 24 mealworms.

### Experimental Sessions

All birds were then assigned to one of four experimental groups (see Table 1), before being given a series of six experimental sessions. In each session a bird was given a randomized series of 12 undefended and 12 defended prey, with the size of each prey type varying among the four experimental groups. The experiment was a 2×2 design in which both the defended and the undefended prey could be either large or small. The groups were named according to the size of the undefended prey and the size of the defended prey, respectively (see Table 1). As in training, a single mealworm was presented every 3 min and each bird had 1 min to attack before it was removed. Undefended and defended prey were made visually distinct from one another by having different coloured backgrounds. These were green and purple paper discs placed in the petri dish beneath the mealworms. The design was balanced within groups, with five birds in each group getting undefended prey on green backgrounds and defended prey on purple backgrounds, and five birds getting undefended prey on purple backgrounds and defended prey on green backgrounds. The six experimental sessions took place across 6 consecutive days, at which point birds appeared to have reached stable asymptotic attack rates on both undefended and defended prey.

### Postexperimental Choice Sessions

On the day after the end of the experimental sessions, we gave each bird a postexperimental choice session, where all mealworms were undefended, to test what cues were being used in its foraging decisions. This consisted of 24 paired presentations in which two individual mealworms were presented singly in two petri dishes placed approximately 10 cm apart in the cage. Birds were given 1 min to select one of the mealworms before both dishes were removed from the cage. Once a bird had made a choice, both dishes were removed immediately. Paired presentations were made every 3 min, as in the previous sessions. All birds received 12 ‘colour-only’ presentations, where they were given the choice between two medium-sized mealworms: one placed on a green and the other on a purple background. They also received 12 ‘size-only’ presentations, where they received a large and a small mealworm placed on white backgrounds in the petri dish. Half the birds received the colour-only presentations first followed by the size-only presentations, and for the remaining birds, the order was reversed. This session enabled us to test whether birds had learned the colour–toxin association, and also to investigate whether size was used in their decision making.

### Data Analysis

As we were interested in the actions of educated predators, we were keen to include only those birds that had learned to discriminate between undefended and defended prey in our analysis. Whether or not birds had learned the discrimination was determined by examining their prey preference in the choice sessions with coloured backgrounds (see above). We were able to determine which birds showed a preference for the colour that was used to signal undefended prey in their experimental sessions. Only birds that chose mealworms on the ‘undefended colour’ in more than 50% of the paired presentations were included in our analysis: 10 birds from the Small–Small group, eight birds from the Small–Large group, eight birds from the Large–Small group and nine birds from the Large–Large group (Table 1).

We determined at which experimental session the groups of birds had reached asymptotic attack rates by running a number of repeated measures ANOVAs (in SPSS 19; SPSS Inc., Chicago, IL, U.S.A.), on the attack data, with prey type and session number as repeated measures. We initially included sessions 1–6, then sessions 2–6, then sessions 3–6, until there was no significant effect of session number. To test whether or not there was an overall effect of prey size on the number of defended prey eaten at asymptote, we then carried out a generalized linear model (GLM) on the data on the numbers of defended prey eaten, with undefended prey size and defended prey size as fixed factors. Finally, we tested whether or not the findings may have been affected by differences in energetic state between groups arising from differences in prey size. We calculated the total prey mass ingested, within a session, at asymptote for each bird and carried out a GLM on the total prey mass ingested, with undefended prey size and defended prey size as fixed factors.

To determine whether or not the birds were using colour signals and/or size differences to discriminate visually between prey types we analysed the data from the postexperimental choice sessions to assess which birds were showing preferences (i.e. choosing one colour/size in more than 50% of the paired presentations).

## Results

**Figure 1:**
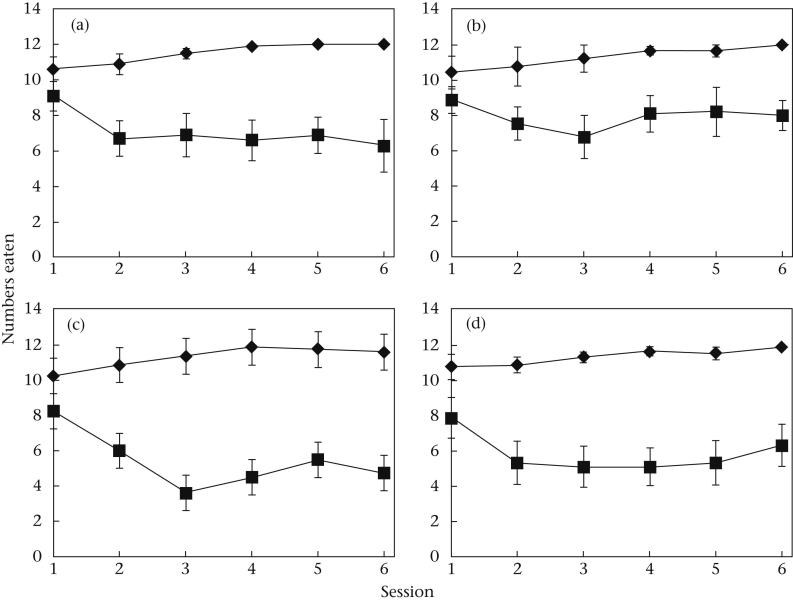
The mean number of undefended prey (diamonds) and defended prey (squares) that were eaten across all sessions in the (a) Small–Small, (b) Small–Large, (c) Large–Small and (d) Large–Large group. Error bars show SEs.

All groups learned to discriminate between undefended and defended prey and reached stable asymptotic attack rates on both undefended and defended prey by session 3 (see Fig. 1), with almost all undefended prey being eaten from this session onwards. We ran a series of repeated measures ANOVAs on the data for both undefended and defended prey in all experimental groups, initially for sessions 1–6, then sessions 2–6 and then sessions 3–6. We found that there was no significant difference in the numbers of undefended or defended prey eaten in any group across sessions 3–6 (repeated measures ANOVA for all groups: 0.16 < *F*_3, 21–27_ < 2.27, 0.10 < *P* < 0.92; Fig. 1) and no significant interaction between prey type and session (0.40 < *F*_3, 21–27_ < 0.88, 0.47 < *P* < 0.75; Fig. 1), but there was a significant difference in the number of undefended and defended prey eaten across sessions 3–6 in all groups (14.65 < *F*_1, 7–9_ < 50.59, <0.0001 < *P* < 0.005; Fig. 1). We concluded that the consumption of undefended and defended prey eaten in a session had reached a stable asymptotic level by session 3 for all groups, and considered our birds to be ‘educated’ at this point. Therefore we conducted all further analyses on data from sessions 3–6.

**Figure 2:**
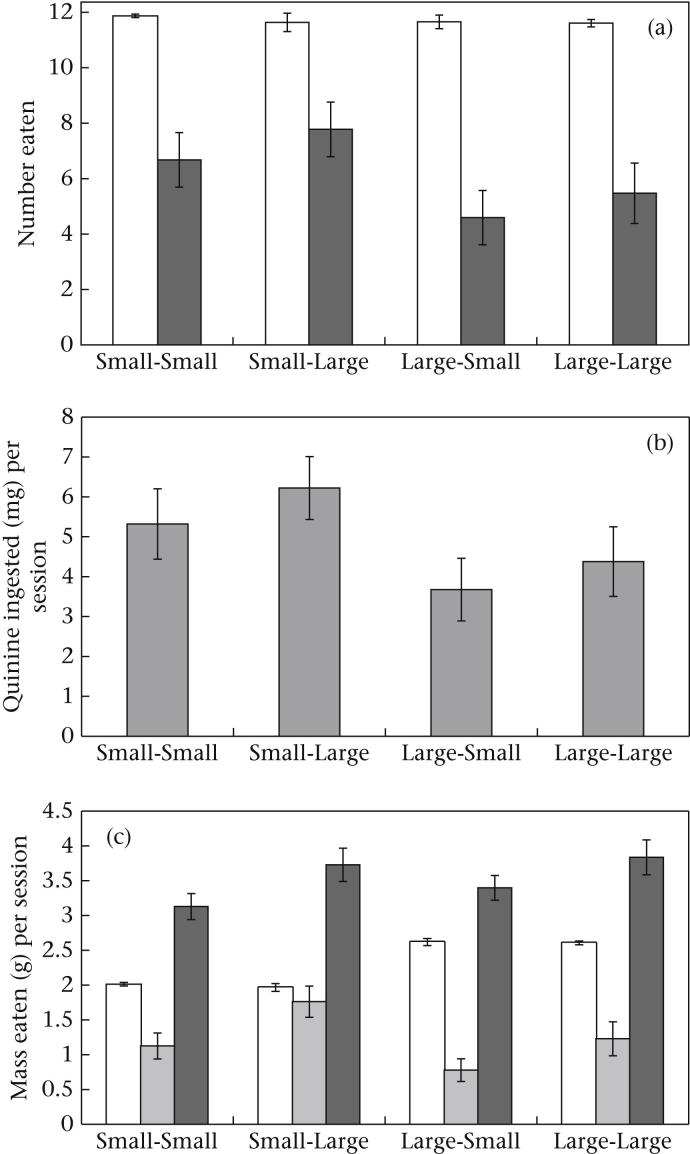
(a) The mean number of undefended prey (white bars) and defended prey (grey bars) eaten; (b) the mean amount of quinine ingested per session at asymptote; and (c) the mean mass of undefended prey (white bars), defended prey (light grey bars) and undefended plus defended prey (dark grey bars) eaten per session at asymptote. Error bars show SEs.

To test our main predictions we compared the mean number of defended prey eaten at asymptote across the different groups. As predicted, we found that the birds given small undefended prey ate a greater number of defended prey than birds given large undefended prey (*F*_1, 32_ = 4.30, *P* = 0.046; see Fig. 2a). However, the size of the defended prey itself had no effect on the number of these prey that were eaten, with birds given small defended prey eating similar numbers to birds given large defended prey (*F*_1, 32_ = 0.876, *P* = 0.36; see Fig. 2a). There was also no significant interaction between the number of undefended and defended prey eaten (*F*_1, 32_ = 0.011, *P* = 0.916). As all defended prey contained the same amount of quinine, these findings mean that the amount of quinine ingested was not affected by the size of the defended prey (*F*_1, 32_ = 0.876, *P* = 0.36; see Fig. 2b), and that birds given small undefended prey ingested more quinine than those given large undefended prey (*F*_1, 32_ = 4.30, *P* = 0.046; see Fig. 2b). Our inability to detect an effect of the size of the defended prey may have been because of the small sample size (observed power = 0.149).

To test for differences in the prey mass eaten by birds in each group, we calculated the total mass of prey ingested in a session (defended and undefended) by the birds at asymptote. We found that the size of the undefended prey had no effect on the total prey mass eaten at asymptote (*F*_1, 32_ = 0.749, *P* > 0.39; see Fig. 2c), but that birds given large defended prey ingested a significantly greater total prey mass than the birds given small defended prey (*F*_1, 32_ = 5.68, *P* = 0.023; see Fig. 2c). Given that mass directly correlates with energy (Finke 2002), it is likely that the birds given large defended prey were in a better energetic state during the test sessions than those given small defended prey.

**Figure 3:**
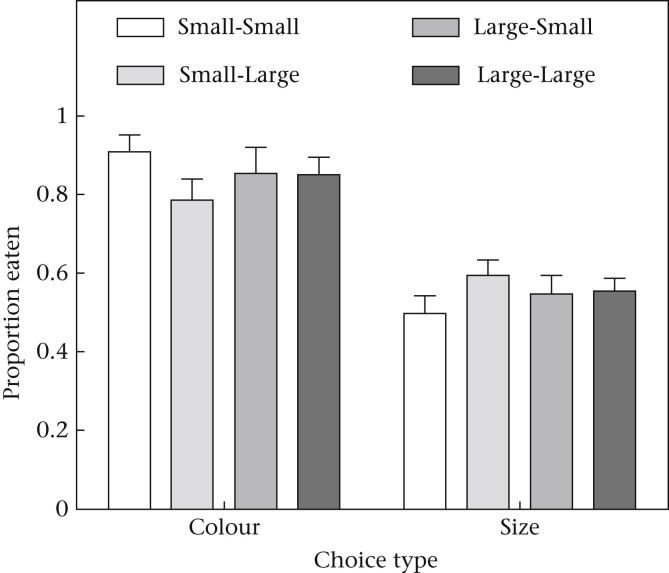
The mean proportion of choices made by each group for medium-sized prey presented on an undefended colour background in the colour choice trials and for large prey in the size choice trials. Error bars show SEs.

Finally, to determine what visual cues the birds were using to make their foraging decisions, we analysed the data from the postexperimental choice session, similarly to previous studies (e.g. Barnett et al. 2007). All birds received 12 presentations of two medium-sized mealworms, where one was placed on the coloured background associated with the undefended prey and the other on a coloured background associated with the defended prey. Testing whether birds had learned to associate colour with the defence level was particularly important for birds in groups in which the size of the undefended and defended prey differed and could have been used as a discriminatory cue. All groups showed a significant preference for medium-sized prey presented on the coloured background that had been previously associated with the undefended prey (one-sample *t* test: all *t* > 6.52; all *P* < 0.001; see Fig. 3). In contrast, when small and large prey were presented together on a white background, none of the groups chose significantly more large prey compared with small prey (all *t* < 2.02; all *P* > 0.08; see Fig. 3). These findings indicate that even when birds had experienced different sized undefended and defended prey, they tended to use colour signals but not size cues when making their decisions in our experiment.

## Discussion

We found clear support for our hypothesis that predators would eat significantly more defended prey and ingest more toxin when undefended prey were small compared with when they were large. However, the size of defended prey had no detectable effect on the number of defended prey eaten, or the amount of toxin ingested. Consequently, the amount of toxin ingested was related to the mass of undefended prey eaten within a session rather than the total mass of prey eaten within a session. These findings indicate that decisions to eat defended prey were not based on a bird's overall energetic state, and that decisions were influenced more by the energetic content of undefended than defended prey. Our results provide novel insights into the mechanisms underlying learning and decision making in avian predators, and have significant implications for the evolution of prey defences.

The fact that birds given large undefended prey ate fewer defended prey than birds given small undefended prey is consistent with the idea that predators eat more aposematic prey (and their mimics) when access to alternative energy supplies is scarce (Brower et al. 1968; Turner & Speed 2001; Kokko et al. 2003; Sherratt 2003). Experiments that have manipulated the abundance of undefended prey have provided only mixed support for this idea (Lindström et al. 2004; Rowland et al. 2010). However, by manipulating the size rather than abundance of undefended prey, our experiment clearly shows that the energy derived from alternative palatable prey are important in determining predation rates on defended prey. In addition, for the first time we are able to elucidate how the energetic content of undefended prey actually influences birds' decisions to eat defended prey. State-dependent models of the evolution of prey defences assume that predators' decisions to eat defended prey are based on the total nutrient intake gained from eating both defended and undefended prey (Kokko et al. 2003; Sherratt 2003; Sherratt et al. 2004). This is because the motivation to acquire energy from defended prey is thought to depend upon a predator's current energetic state, determined by its overall energetic and nutritional intake. However, this appears not to be the case. Groups that received large defended prey ate more mealworm mass and were therefore in a better state (Fig. 2c), yet they did not eat fewer defended mealworms (Fig. 2a). Therefore, we can conclude that the energy gained from undefended prey seems to be more important in determining attacks on defended prey than that gained from all prey combined.

This has significant implications for understanding the selection pressures acting on aposematic prey. First, predation on aposematic prey is predicted to increase when the size and energy available from undefended prey decrease (Kokko et al. 2003; Lindström et al. 2004; Sherratt et al. 2004), and consequently the benefits to being aposematic will probably vary across the year. For example, when the availability of energy from lepidopteran larvae decreases in the late summer and autumn in temperate climes, avian predators need to turn to alternative sources of food (Ide 2006). Our findings suggest that this could have important consequences for aposematic prey, and could explain seasonal changes in defensive coloration and strategy in insects: for example, why the shieldbug, *Graphosoma lineatum*, is cryptic in the autumn, when alternative prey may be scarce, but aposematically coloured in the spring (Johansen 2011), when alternative prey are likely to be more abundant; and why overwintering seven-spot ladybirds, *Coccinella septempunctata*, aggregate from early autumn onwards (Barron & Wilson 1998). Aggregations of aposematic prey are thought to increase the efficacy of the visual warning signals and speed up avoidance learning in predators (Gagliardo & Guilford 1993; Gamberale & Tullberg 1996). Many species in fact show ontogenetic or seasonal shifts in their defensive strategy (Booth 1990), and this could in part be explained by changes in the energy available (or perhaps even the specific nutrients available: see Turner & Speed 2001; Mayntz et al. 2005) from undefended prey. Second, our results suggest that changes in the life history strategies of undefended and cryptic prey could also significantly affect the predation of aposematic prey. For example, if the likelihood of being predated before reaching reproductive age is high, undefended prey may be selected to complete their life cycle more quickly (Werner 1986; Rowe & Ludwig 1991). This change in life history strategy is commonly associated with a reduction in body size (Arendt 1997 and references therein) that could lead to an increase in the predation pressures acting on aposematic prey.

We were surprised that the size of the defended prey had no effect on the birds' decisions to eat defended prey. This is in contrast to studies of grazing animals (including crustaceans, fish and mammals), in which the amount of toxic food eaten increases with the energetic and overall nutritional value of that food (Duffy & Paul 1992; Cruz-Rivera & Hay 2003; Villalba & Provenza 2005). At least in the case of mammalian herbivores, a higher nutritional value is thought to enable them to invest more heavily in detoxification processes and ingest more toxin (Marsh et al. 2005; Villalba & Provenza 2005). Our birds did not appear to use the additional energy content of the large defended prey in this way.

There are several explanations why the size of defended prey was not used in predators' decisions to eat defended prey. First, birds may not have learned about the energetic content of the defended prey. This could have occurred because the quinine impaired or slowed down the birds' abilities to learn about the energetic value of the defended prey. Although we know little about the toxic effects of quinine, or whether it affects the way in which predators learn about the energetic and nutritional value of prey, there are a number of reports of other toxins having negative effects on learning (Dudai et al. 1987; Levin et al. 2003). Second, if birds did learn about the energetic value of defended prey, their decisions may have been more heavily influenced by their knowledge about the toxin content compared with the energy content of the defended prey. The ‘negativity effect’ occurs when an individual weights negative information more heavily than positive information in their decision making, and is thought to be an evolutionary adaptation to ensure that animals remain vigilant for negative events and stimuli to avoid undesirable outcomes (Peeters & Czapinski 1990; Taylor 1991). Finally, it may have been that the difference in size between our small and large defended prey was too small to allow birds to gain sufficient energy to detoxify additional amounts of toxin. This perhaps suggests that increasing body size may not be as costly for aposematic prey as it is for cryptic prey, as predators will not eat more aposematic prey unless they are large enough to offset the costs of eating more toxin. Therefore, it remains a possibility that more pronounced differences in the size of defended prey could affect predators' decisions to eat them. Notably, although our study was designed to test specifically the effect of the body size of toxic prey on the foraging choices of predators, manipulating the toxin content or prey relative to body size would be an interesting future line of study.

Finally, our results suggest that size may not be as important in foraging decisions as coloration is. Our simultaneous choice trials showed that birds had learned to associate the coloration but not the size of the prey with the toxicity. This suggests that the coloration was a more salient cue that overshadowed the birds' abilities to learn about differences in size between our two prey types (Pavlov 1927). If wild birds respond to live aposematic prey in the same way as the birds in our experiment responded to the artificial prey, their behaviour will have implications for the evolution of Batesian mimicry, where mimics are often smaller than their models (Marples 1993). A recent comparative study suggested that the mimicry of hymenopteran models by palatable hover flies was less accurate the smaller the hover fly mimic was (Penney et al. 2012). Therefore, if predators are using size as a cue to discriminate between models and mimics, mimicry may start to become less advantageous to the mimic with decreasing body size. Our results, however, argue against this interpretation, and suggest that Batesian mimics could differ in size from their model without predators necessarily using that information to discriminate between them. This could be particularly true in cases where the warning signal is highly conspicuous and salient to the predator. It is possible that large aposematic prey with highly conspicuous and salient warning signals could be mimicked by much smaller species if the colour signal overshadows any size cue in the learning process.

In conclusion, our results highlight the need to consider the mechanisms underlying foraging decisions by educated predators. Although there is an extensive and valuable body of theoretical and empirical work exploring how predators learn to avoid aposematic prey (e.g. Friedlander 1976; Gittleman et al. 1980; Guilford 1988; Alatalo & Mappes 1996), we argue that how predators trade off the costs of eating toxins with the benefits of energy intake should be considered as an equally potent selection pressure. Our study did not detect an effect of prey size on birds' foraging decisions towards defended prey as we originally expected, and the fact that predators' decisions to eat defended prey were more affected by changes in the size of undefended than defended prey raises interesting questions about how the selection pressures acting on undefended prey can influence predation pressures on defended prey. Our results highlight the need to understand the mechanisms underlying predator decision making in the wider context of the foraging opportunities available to them in order to understand fully the selection pressures acting on aposematic prey.

## Figures and Tables

**Table 1:** The numbers and size of each prey type given to birds, and the number of males and females, in each of the four experimental groups

Experimental group	Number of males	Number of females	Undefended prey	Defended prey
Small–Small	5 (5)	5 (5)	12 Small (0.15–0.18 g)	12 Small (0.15–0.18 g)
Small–Large	5 (4)	5 (5)	12 Small (0.15–0.18 g)	12 Large (0.23–0.25 g)
Large–Small	5 (4)	5 (4)	12 Large (0.23–0.25 g)	12 Small (0.15–0.18 g)
Large–Large	5 (4)	5 (5)	12 Large (0.23–0.25 g)	12 Large (0.23–0.25 g)

Numbers in parentheses refer to numbers that showed a preference for prey on the undefended prey colour in the choice session.
